# Authentic Assessment of Social–Emotional Development in Early Childhood: A Scoping Review

**DOI:** 10.3390/brainsci16080806

**Published:** 2026-07-30

**Authors:** Yuyan Xia, Yaoying Xu, Lin Zhu, Jun Ai, Chin-Chih Chen

**Affiliations:** 1 Department of Language Science and Technology, Faculty of Humanities, The Hong Kong Polytechnic University, Hong Kong, China; 2 Department of Counseling and Special Education, Virginia Commonwealth University, Richmond, VA 23284, USA; 3 Department of Special and Early Education, College of Education, Northern Illinois University, DeKalb, IL 60115, USA; 4 Department of Theory and Practice in Teacher Education, University of Tennessee, Knoxville, TN 37996, USA

**Keywords:** authentic assessment, social–emotional development, early childhood, scoping review, naturalistic observation, sociocultural theory, interpretive assessment, 0–8 years

## Abstract

**Highlights:**

**What are the main findings?**

- Twenty-nine authentic SED assessment tools were identified across 33 studies, with naturalistic observation dominating (69.7%) while portfolio and curriculum-embedded approaches remained markedly under-represented.
- “Authenticity” is operationalized across ecological, participatory, and cultural dimensions that are rarely theorized in an integrated manner, revealing significant conceptual fragmentation.

**What are the implications of the main findings?**

- Substantial gaps in infant–toddler and primary-grade coverage, geographic diversity (57.6% U.S.-based), and cultural adaptation call for targeted tool development and cross-cultural validation research.
- The integrated sociocultural and interpretive assessment framework developed in this review offers an analytic model that may support equitable, contextually responsive SED assessment across the 0–8 continuum.

**Abstract:**

**Background/Objectives**: Authentic assessment—the systematic observation of naturally occurring social–emotional behavior by familiar caregivers—is increasingly mandated by international early childhood frameworks, yet the field lacks a comprehensive tool inventory, conceptual clarity on “authenticity,” and synthesized evidence on contextual coverage. This scoping review maps authentic social–emotional development (SED) assessment tools for children aged 0–8, examines how authenticity is conceptualized, and identifies gaps in cultural adaptation, disability inclusion, and age-range coverage. **Methods**: Following the Arksey and O’Malley framework enhanced by Levac et al. and JBI methodology, six databases (PsycINFO, ERIC, Education Source, MEDLINE, Scopus, Web of Science) were searched for English-language peer-reviewed publications (2006–2026), supplemented by hand-searching and forward citation tracking. Inclusion criteria targeted children aged 0–8 assessed via authentic modalities (naturalistic observation, play-based assessment, portfolio, performance-based tasks, curriculum-embedded measurement) in any global setting. Data charting used an integrated sociocultural and interpretive assessment framework. Reporting followed PRISMA-ScR. **Results**: Thirty-three studies yielded 43 instruments, 29 meeting authentic assessment criteria. Naturalistic observation predominated (23 studies; 69.7%), followed by play-based assessment (10; 30.3%); portfolio and curriculum-embedded approaches were under-represented. Most studies originated from the United States (57.6%) and targeted preschoolers (3–5 years; 75.8%), with thin coverage of infants/toddlers and primary-grade children. “Authenticity” was operationalized across ecological, participatory, and cultural dimensions rarely integrated theoretically. **Conclusions**: This review provides the first systematic landscape map of authentic SED assessment for children aged 0–8, confirming feasibility and diversity while revealing gaps in age coverage, geographic reach, and cultural adaptation. The integrated framework offers a potentially transferable analytic model, and findings can inform equitable, contextually responsive assessment practice and policy.

## 1. Introduction

### 1.1. Global Context and Policy Landscape

Globally, an estimated 250 million children under age five in low- and middle-income countries are at risk of not reaching their developmental potential. In high-income countries, 10–20% of children show clinically significant social–emotional difficulties before school entry [1]. The economic burden of mental ill-health is estimated to amount to more than 4% of GDP across OECD countries, corresponding to several trillion US dollars annually [2]. These developmental and economic concerns have increased policy attention to early identification and assessment. Early childhood initiatives such as Head Start in the United States, the Early Years Foundation Stage in England, and the Early Years Learning Framework in Australia all emphasize observation-based and contextually embedded assessment. These approaches align with the broader movement toward authentic assessment in early childhood.

Research output has similarly growth publications in the 2016–2026 decade have at least doubled compared to 2006–2015, driven by observational technology, policy mandates for cultural responsiveness, and inclusive practices for children with disabilities. However, a central tension remains. The field increasingly values authentic assessment, but there is limited consensus on what “authenticity” means in practice. It is also unclear whose perspectives count as evidence and how assessment data should inform pedagogical action.

### 1.2. Conceptual Definitions

Social–emotional development (SED), as defined by CASEL (2020) [3], encompasses self-awareness, self-regulation, social awareness, relationship skills, and responsible decision-making, constructs further positioned by the WHO Nurturing Care Framework and UN SDG Target 4.2 within a global mandate for quality early childhood development. In this review, authentic assessment refers to the systematic recording of children’s naturally occurring behaviors during daily routines by familiar and knowledgeable caregivers or educators. This definition includes naturalistic observation, play-based assessment, portfolio documentation, performance-based tasks, and curriculum-based measurement. Unlike standardized norm-referenced testing (e.g., BASC-3, SSIS), which sacrifices ecological validity and cultural responsiveness for psychometric precision, or informal teacher judgment, which lacks systematic documentation and evidentiary structure, authentic assessment bridges ecological validity and systematic documentation. The social–emotional domain also presents specific challenges for authentic assessment. SED constructs are relational and culturally shaped. The 0–8 age span crosses infant–toddler care, preschool, and early primary schooling. In addition, observations from teachers, families, and other informants may not always align.

### 1.3. Research Rationale and Gaps

Despite two decades of progress, including narrative and systematic reviews, tool development, policy syntheses, and equity scholarship, the field has not yet systematically mapped the intersection of authentic assessment and SED across the full 0–8 age range. Three prioritized gaps remain: Gap 1 (Methodological): no review has charted the full landscape of authentic SED tools, their characteristics, and psychometric properties; Gap 2 (Conceptual): “authenticity” is used with incompatible meanings across ecological, participatory, and cultural dimensions; Gap 3 (Contextual): evidence on cultural adaptation, disability inclusion, and coverage across the 0–8 continuum remains sparse. Two trade-offs are particularly acute: the tension between ecological validity and psychometric rigor, and between cultural universalism and cultural particularism in defining SED constructs.

### 1.4. Research Questions and Scope

Based on the three gaps identified above, this scoping review aims to address the following research questions, guided by the framework of Arksey and O’Malley [4] as enhanced by Levac, Colquhoun, and O’Brien [5] and the JBI methodology [6]:

**RQ1 (responding to Gap 1, Methodological):** What authentic assessment tools have been used to measure SED in children aged 0–8 years, and what are their key characteristics?

**RQ2 (responding to Gap 2, Conceptual):** How is “authenticity” conceptualized and operationalized across the included studies, and what patterns of convergence and divergence are evident in the ecological, participatory, and cultural dimensions of authenticity?

**RQ3 (responding to Gap 3, Contextual):** What gaps exist in the current evidence base across the ages 0–8 continuum, cultural and linguistic adaptation, inclusion of children with disabilities or developmental delays, SED sub-domain representation, and multi-informant design—and how has the research landscape changed over time?

This review focuses on children aged 0–8 years, a range that corresponds to the internationally recognized “early childhood” period as defined by the UN Committee on the Rights of the Child (General Comment No. 7, 2005) and encompasses the three institutionally distinct sectors: infant–toddler care, pre-kindergarten/preschool, and early primary schooling, whose fragmentation constitutes a central concern of this review. The review encompasses studies published in English between 2006 and 2026, a twenty-year window selected to capture both the foundational period of authentic assessment research (2006–2015) and the more recent decade of accelerated growth and policy integration (2016–2026). It includes studies conducted in any setting (home, childcare, preschool, primary school, clinic) and in any country, reflecting the global scope of the policy mandate. The Population–Concept–Context (PCC) framework is summarized in Table 1.

## 2. Related Literature

### 2.1. Theoretical Foundations of Authentic Assessment

Authentic assessment of social–emotional development in early childhood draws from developmental psychology, educational measurement, early childhood pedagogy, and cross-cultural developmental science. These traditions emphasize different aspects of assessment, including individual development, psychometric quality, observation and documentation, professional judgment, and cultural variation. As a result, the same child behavior may be conceptualized, measured, and interpreted differently depending on the disciplinary lens.

The term “authentic assessment” entered the educational lexicon through the work of Wiggins [7], who argued that assessment tasks should replicate the challenges and standards of performance that matter in real-world contexts rather than relying on proxy indicators such as multiple-choice items. In early childhood, Bagnato [8] and Neisworth and Bagnato [9] adapted this idea to developmentally appropriate assessment. They defined authentic assessment as the systematic recording of young children’s naturally occurring behaviors in daily routines by familiar and knowledgeable caregivers. This definition can be decomposed into four constitutive elements: (a) *systematic recording*, distinguishing authentic assessment from informal impression; (b) *over time*, implying longitudinal documentation rather than single-occasion measurement; (c) *naturally occurring behaviors in daily routines*, specifying ecological context; and (d) *by familiar and knowledgeable caregivers*, positioning the assessor as an insider participant rather than an external examiner.

Three theoretical traditions have shaped the scholarly understanding of authentic SED assessment, each contributing a different explanatory lens organized from the micro-level (individual development) to the macro-level (societal systems). First, developmental-constructivism [10,11] conceptualizes the child as an active constructor of social understanding, implying that assessment should capture the child’s reasoning and meaning-making rather than merely catalog observable behaviors. This tradition has informed portfolio and learning-story approaches that privilege the child’s perspective and prioritize developmental progression. Second, sociocultural theory [12,13] reframes social–emotional competence as situated within social practices and cultural communities, emphasizing the role of more experienced partners, cultural tools, and institutional contexts. From this perspective, competence is a property of person-in-context rather than person-alone, and assessment must be made of the relational and cultural ecology within which development unfolds. Third, critical and post-structural perspectives [14,15] question the normalizing assumptions embedded in developmental frameworks and assessment practices, drawing attention to how definitions of “social competence” may reproduce dominant-culture norms and marginalize non-dominant ways of being. This tradition highlights questions of power, equity, and the politics of representation in assessment.

These traditions offer complementary but incomplete perspectives. Developmental constructivism foregrounds children’s agencies and developmental change. Sociocultural theory emphasizes relationships, routines, and cultural context. Critical perspectives highlight power, equity, and representation. However, no single tradition fully captures the ecological, cultural, and interpretive complexity of authentic SED assessment. This limitation is addressed by the integrated framework introduced in Section 3.

### 2.2. Authentic Assessment in Social–Emotional Development

Authentic assessment of SED presents several practical challenges. Developmental-constructivist approaches have informed learning stories in Aotearoa New Zealand and Reggio Emilia-inspired documentation in Italy, but rich narrative documentation can be difficult to translate into structured evidence for cross-sector communication and accountability systems. Sociocultural approaches have supported multi-informant assessment models, yet the reconciliation of discrepant perspectives, such as differences between educator and parent observations, remains underspecified. Critical perspectives have identified cultural assumptions embedded in tools such as the Ages and Stages Questionnaires: Social–Emotional (ASQ), but fewer studies provide concrete procedures for culturally responsive assessment beyond participatory design.

Several lines of scholarship have attempted to address these challenges. Structured observation protocols, such as the Devereux Early Childhood Assessment (DECA) and the Infant–Toddler Social Emotional Assessment (ITSEA), aim to improve reliability while retaining ecological validity. Technology-enhanced documentation, including video-assisted coding, digital portfolios, and tablet-based observation apps, has been used to support more systematic recording and sharing of assessment evidence. Hybrid models combine authentic observation with structured rating scales or curriculum benchmarks, while participatory design approaches involve families and cultural communities in assessment development or interpretation. These efforts show promise, but they also involve trade-offs: greater standardization may reduce ecological and cultural responsiveness, whereas more participatory approaches may require time and resources that are not always available in early childhood settings.

The conditions under which these tools are used well, including assessor preparation, cultural competence, relational quality, and institutional supports, have received comparatively little systematic attention. Whether the challenges of authentic SED assessment are primarily technical or also interpretive is therefore treated in this review as an open empirical question, taken up in the Discussion section (Section 6.3) in light of the charted evidence.

The social–emotional domain introduces specificities that are not adequately captured by generic models of authentic assessment. Unlike literacy or numeracy, where there are relatively clear behavioral referents for competence, SED constructs such as “empathy,” “self-regulation,” and “social competence” are *culturally constituted* (what counts as prosocial behavior varies across cultural communities), *relationally enacted* (a child’s social competence emerges differently with different interactional partners and in different emotional climates), and *context-dependent* (the same child may demonstrate self-regulation in one setting and not another). This means that the assessor is not simply documenting a pre-existing, stable reality but actively constructing an interpretation through culturally inflected lenses, a reflexivity that standardized assessment is designed to minimize but that authentic assessment must embrace and manage.

How assessors manage this interpretive work in practice, and what role the educator assessor plays in shaping assessment quality, are addressed in the Discussion (Section 6.3 and Section 6.4) as interpretations emerging from the reviewed evidence.

### 2.3. Methodological Limitations and Research Gaps

A small but growing body of qualitative research further suggests that practitioners differ systematically in their assessment orientations, for example, in whether observation is used primarily to identify problems relative to normative benchmarks or to document children’s emerging strengths and relational contexts. The practice-level significance of these differences is examined in Section 6.2 in light of the review findings.

Theory-driven (deductive) typologies have also been proposed. Bagnato’s [8] distinction among “authentic,” “conventional,” and “convergent” assessment provides a widely cited classification, while Shepard’s [16] framework differentiates between “assessment of learning,” “assessment for learning,” and “assessment as learning.” These deductive frameworks offer systematic organizing categories, but they have specific limitations: Bagnato’s typology focuses primarily on the format of the tool (what data are collected) rather than the interpretive practice of the assessor (how data are used); Shepard’s framework was developed for school-age contexts and does not fully accommodate the developmental specificities of the 0–8 range; and neither typology adequately captures the cultural dimension of assessment practice. The complementarity between inductive insights (from practitioners’ experience) and deductive frameworks (from theoretical models) suggests that a systematic mapping of the literature, one capable of detecting both theoretically predicted and empirically emergent patterns, would add significant value.

Synthesizing the analysis above, three research gaps are identified and ranked by priority. Gap 1 (Highest Priority—Methodological): No existing review has systematically mapped the full landscape of authentic SED assessment tools for children aged 0–8, charting their characteristics, psychometric properties, and coverage patterns. This gap is ranked highest because the panoramic map is a prerequisite for addressing the subsequent two gaps: without knowing what exists, it is impossible to identify where conceptual fragmentation occurs or where contextual evidence is missing. Gap 2 (Conceptual/Explanatory): The conceptual relationship among ecological, participatory, and cultural dimensions of “authenticity” has not been systematically analyzed, leaving practitioners and researchers operating with implicit and potentially inconsistent definitions of what makes an assessment “authentic.” Gap 3 (Contextual/Applied): Evidence on cultural and linguistic adaptation, inclusion of children with disabilities, and coverage across the full 0–8 age continuum remains sparse, the gap most consequential for equity.

The limitations identified above collectively point to the need for a comprehensive, systematic mapping of the field. Individual studies and narrative reviews have addressed important aspects, but the overall landscape remains obscured by heterogeneity of definitions, methods, contexts, and disciplinary assumptions. A scoping review, with its capacity to chart breadth, identify patterns across heterogeneous evidence, and detect gaps without requiring methodological homogeneity, is the methodology best suited to this task.

## 3. Theoretical Framework

This review adopts an integrated analytical lens combining sociocultural theory and interpretive assessment theory. Sociocultural theory emphasizes that social–emotional competence is distributed, culturally mediated, and co-constructed in interaction (Vygotsky [12], Rogoff [13], Fleer [17]). Interpretive assessment theory frames assessment as an interpretive process shaped by selective attention, evidential reasoning, and negotiation across informants (Moss [18], Shepard [16], Delandshere [19]). It therefore helps explain how assessment evidence is noticed, interpreted, discussed, and translated into action. Together, these two perspectives guided both the conceptual framing and the synthesis of included studies.

To operationalize this framework, seven a priori analytical categories were embedded in the data-charting form (Section 4.4). From the indicator space (sociocultural axis), three categories captured what each tool measured and under what conditions: cultural and institutional context (whether and how the tool addressed cultural variation in social–emotional expression and expectation), interactional quality (whether the tool assessed competence as a property of person-in-context, for example in adult–child or peer interactions), and performance conditions (whether supported as well as independent performance was documented). From the practice space (interpretive axis), four categories captured how each study addressed the assessment process: selective attention (what observers are directed to notice), evidential reasoning (how observations are scored or interpreted), dialogic negotiation (how discrepancies among informants are handled), and pedagogical action (how results inform practice). Each included study was coded against these seven categories during data charting, with disagreements resolved by discussion. The indicator-space categories directly informed RQ2 (conceptualizations of authenticity) and the cultural-adaptation component of RQ3, whereas the practice-space categories informed the charting of tool characteristics under RQ1 and structured the synthesis of interpretive practices presented in the Discussion (Section 6.2, Section 6.3 and Section 6.4).

## 4. Methods

### 4.1. Protocol

This scoping review followed PRISMA-ScR guidelines [20] and JBI methodology [6], with the protocol developed a priori, which guided the eligibility criteria, search strategy, study selection, and data charting procedures, but the protocol was not prospectively registered. Study screening and data management were conducted using Covidence [21]. The completed PRISMA-ScR checklist is available as Supplementary Table S1.

### 4.2. Eligibility Criteria

Studies were included if they involved children aged 0–8 years and described, developed, validated, or applied at least one authentic assessment tool, operationalized as naturalistic observation, play-based assessment, portfolio documentation, performance-based tasks, or curriculum-embedded measurement; and they targeted one or more SED constructs (e.g., self-regulation, social competence, empathy, prosocial behavior). Any geographic setting was eligible; only peer-reviewed English-language articles published between January 2006 and January 2026 were considered. Studies relying exclusively on standardized norm-referenced instruments, intervention-outcome studies that did not describe the assessment tool in sufficient detail, and non-empirical publications were excluded. Where exact participant ages were not reported, grade or program descriptors were used to confirm eligibility [22].

One exception to the publication window was permitted a priori: records identified through hand searching or forward citation tracking that predated 2006 were eligible if they reported the original validation of an authentic assessment instrument that remained in active use within the review period. One study, Gagnon, 2004 [23], was included on this basis (see Section 5.1 and Section 5.2.1 and the note to Table 2).

### 4.3. Search Strategy

Systematic searches were conducted across six databases: PsycINFO, ERIC, and Education Source (via EBSCOhost), MEDLINE (via PubMed), Scopus, and Web of Science. The strategy, developed with a research librarian and pilot-tested in PsycINFO, combined three concept blocks: authentic/performance-based assessment terms, child/early childhood terms, and SED-related terms, joined by AND (see Supplementary S1 for the full string). Reference lists of included studies and relevant reviews were hand-searched, and forward citation tracking of seminal tool-development articles was conducted.

### 4.4. Study Selection and Data Charting

Retrieved records were de-duplicated in Covidence and screened in two stages (title–abstract, then full text) by two independent reviewers; discrepancies were resolved by discussion or a third reviewer. Inter-rater reliability (Cohen’s κ) was calculated at each stage, with a calibration pilot of 50 records (target κ ≥ 0.80). A data charting form, developed a priori and pilot-tested on 10 studies, captured bibliographic details, study design, participant characteristics, tool characteristics (target age range, SED constructs assessed, administration format, assessment setting, informant/rater, and reported psychometric properties), and publication period (Period A: 2006–2015; Period B: 2016–2026). Charting was performed independently by two reviewers for the first 20% of studies, with a random 10% sample verified thereafter. In the tables and text, each study is referred to by the first author’s last name and year (e.g., Gagnon, 2004).

To distinguish authentic assessment tools from supplementary or standardized measures, three decision rules derived from the four constitutive elements of the operational definition (Section 2.1) were applied during charting: (a) the tool had to capture naturally occurring behavior in daily routines or familiar settings, rather than examiner-elicited performance in decontextualized testing situations; (b) the informant or observer had to be a familiar adult or an observer documenting behavior in the child’s natural context; and (c) scoring had to be grounded in documented observation rather than global normative judgment alone. Tools meeting all three rules were catalogued as authentic assessment instruments; standardized norm-referenced questionnaires and diagnostic instruments used as criterion, comparison, or supplementary measures were not. The most common ambiguous case, teacher or parent completed rating scales, was resolved by examining whether the scale content referenced observable behavior in naturally occurring routines (classified as authentic, e.g., PIPPS-T) or required global normative comparisons detached from a specified observation context (classified as supplementary). Ambiguous classifications were resolved by discussion between the two reviewers, with the third reviewer consulted where necessary. The complete study-level extraction for all 33 studies is provided in Supplementary Table S2.

### 4.5. Synthesis

Data were synthesized using descriptive quantitative summaries (frequency counts and percentages by period, country, age group, format, and SED construct) and thematic narrative synthesis organized around the three research questions. Although PRISMA-ScR does not require critical appraisal of included sources, a descriptive appraisal of psychometric evidence was incorporated because RQ1 explicitly asks about tool characteristics, of which psychometric support is one dimension. Using an adapted COSMIN-informed checklist [24], five properties were recorded for each tool: internal consistency, inter-rater reliability, test–retest reliability, structural validity, and criterion or convergent validity; this characterized the evidence base but did not determine study inclusion. A visual evidence map [25] was produced to display the distribution of tools across age groups, SED domains, and levels of psychometric support (see Supplementary Table S1 for the PRISMA-ScR checklist and Supplementary Table S2 for the full data extraction table).

## 5. Results

### 5.1. Selection of Sources of Evidence

**Figure 1:**
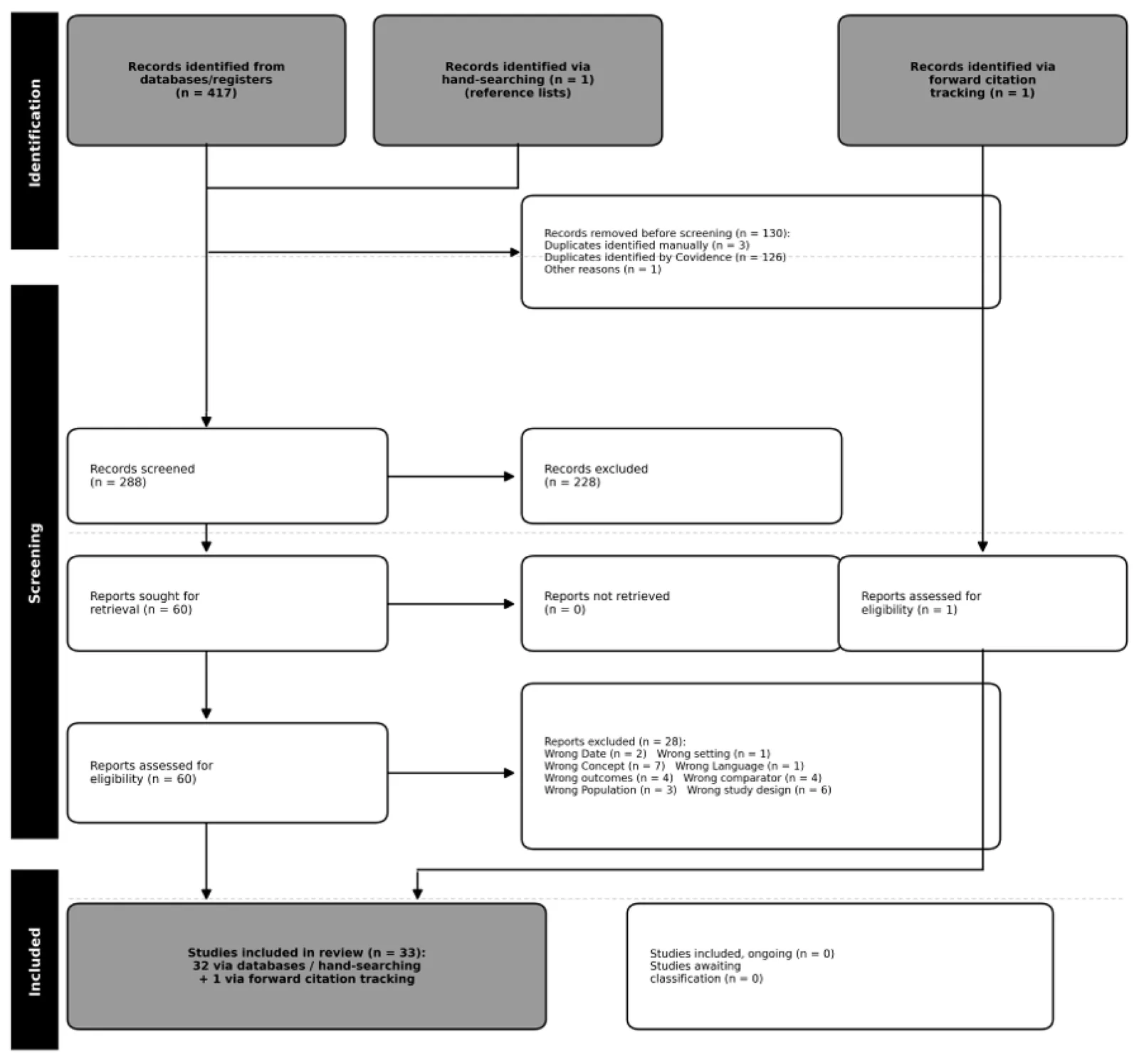
PRISMA-ScR Flow Diagram.

The systematic search of six databases (PsycINFO, ERIC, Education Source, MEDLINE, Scopus, and Web of Science) returned 417 records, supplemented by one record, Gagnon, 2004 [23], was identified through hand-searching of reference lists of included studies and relevant reviews (see Section 4.2). After de-duplication in Covidence, 288 unique records were screened at the title–abstract stage; 228 were excluded as not meeting eligibility criteria, leaving 60 for full-text review. At the full-text stage, 28 articles were excluded (most commonly because they used only standardized norm-referenced instruments, lacked sufficient detail on the assessment tool, or were non-empirical publications), yielding 32 included studies. One further study (Downer, 2010 [26]) was subsequently identified through forward citation tracking of seminal tool development articles and met all eligibility criteria on full-text assessment. The final sample therefore comprised 33 studies: 32 identified through database searching and hand-searching, and 1 through forward citation tracking. Inter-rater agreement (Cohen’s κ) was 0.85 at title–abstract and 0.90 at full text, both above the pre-specified threshold of 0.80. The complete flow is summarized in Figure 1.

### 5.2. Characteristics of Sources of Evidence

#### 5.2.1. Publication Year and Temporal Distribution

The 33 included studies were published between 2006 and 2026, spanning the full 20-year review window. Full charted data for each included study are provided in Supplementary Table S2. When divided into two periods—Period A (2006–2015) and Period B (2016–2026)—15 studies (45.5%) fell in Period A and 18 studies (54.5%) fell in Period B, reflecting a moderate increase in publication volume in the most recent decade (see Table 2).

**Table 2:** Publication Year and Temporal Distribution of Included Studies.

Year	Studies	Period
2004	Gagnon	A
2007	Wimpory	A
2009	Rimm-Kaufman	A
2010	Bulotsky-Shearer; Meisels; Downer	A
2011	Moreno	A
2012	Razza	A
2013	Herndon	A
2014	Hughes; Gower	A
2015	Delvecchio; Kurki; Lambert; Alquraini	A
2017	Hernández	B
2018	Uyanık; Sutherland; Giske	B
2019	Sahin Asi; Ahmad	B
2020	Santos; Jaggy	B
2021	Hamaidi; Hanish; Bauminger-Zviely; Qiu	B
2022	Alamos; Silva Moreira; Sedem; Mathis	B
2023	Lasch	B
2024	Boise	B

Note. Throughout this article, studies are referred to by the first author’s last name and year (e.g., Gagnon). Gagnon (2004 [23]) predates the 2006–2026 database search window and was retained through hand-searching and forward citation tracking, per the a priori exception described in Section 4.2.

Within Period A, publications were relatively evenly distributed (1–4 per year), whereas Period B showed a concentration in 2021 (*n* = 4) and 2022 (*n* = 4), suggesting a recent acceleration of interest. Notably, no studies in the final sample were published in 2016, possibly reflecting either fluctuations in the research cycle or studies captured by the search but excluded at screening. One study (Gagnon, 2004 [23]) falls outside the 2006–2026 database search window and was retained because it was identified through forward citation tracking as an original validation of an authentic peer-play assessment instrument (PIPPS) that remained actively used during the review period. One additional study (Downer, 2010 [26]) within the search window was identified through forward citation tracking of seminal tool-development articles, rather than through the original database search. Downer et al. (2010) [26] reports the initial psychometric validation of the inCLASS, an individualized naturalistic observation system for preschoolers’ classroom interactions, and is the foundational reference for the two subsequent inCLASS-based studies already included (Sutherland, 2018 [27]; Alamos, 2022 [28]).

#### 5.2.2. Geographic Distribution

The United States accounted for more than half the studies (*n* = 19, 57.6%). The remaining 14 studies were distributed across 12 countries: Turkey (*n* = 2), Portugal (*n* = 2), and one study each from Italy, New Zealand, Jordan, Finland, Sweden, Switzerland, the United Kingdom, Israel, Canada, and Norway. No studies from Africa, South or Southeast Asia, Latin America, or the Asia-Pacific region (beyond New Zealand and Israel) were included, signaling a substantial geographic gap (see Table 3).

#### 5.2.3. Study Design

The overwhelming majority of studies employed a quantitative design (*n* = 29, 87.9%). Two studies used a qualitative approach (Kurki, 2015 [51], using qualitative content analysis of video observations; Bauminger-Zviely, 2021 [55], using observational coding in natural settings). One study adopted a mixed-methods design (Sahin Asi, 2019 [45]), and one was classified as “Other: Quantitative” (Boise, 2024 [43], employing a cross-lagged panel design across two years).

#### 5.2.4. Target Age Groups

Studies targeted children at various points across the 0–8 age range. For analytic purposes, studies were coded into four overlapping age bands based on the reported age ranges of participants (see Table 4):

The preschool age band (3–5 years) was by far the most heavily represented, with 25 studies (75.8%) including children in this range. Coverage was notably thin for infants under 12 months (only Razza, 2012 [33]; Lasch, 2022 [42]; Ahmad, 2019 [49], with data beginning at 8–9 months) and for primary school children aged 6–8 years (only Kurki, 2015 [51], and Silva Moreira, 2022 [47], explicitly targeted this range).

#### 5.2.5. Population Characteristics

The majority of studies included typically developing children (*n* = 26, 78.8%). Seven studies included children with disabilities or special needs (Alquraini, 2015 [37]; Boise, 2024 [43]; Sedem, 2022 [52]; Wimpory, 2007 [54]; Bauminger-Zviely, 2021 [55]; Lambert, 2015 [36]; Sutherland, 2018 [27]), and eight studies focused on or included at-risk populations defined primarily by low socioeconomic status (Gagnon, 2004 [23]; Bulotsky-Shearer, 2010 [30]; Alamos, 2022 [28]; Boise, 2024 [43]; Hanish, 2021 [39]; Lambert, 2015 [36]; Moreno, 2011 [32]; Sutherland, 2018 [27]). Some studies included multiple population groups (see Table 5).

### 5.3. Assessment Tools Identified and Catalogued

#### 5.3.1. Overview

Across the 33 included studies, a total of 43 distinct assessment tools were identified that were used to measure or relate to young children’s social–emotional development. Of these, 29 tools were classified as authentic assessment instruments (i.e., they assess children through naturalistic observation, play-based procedures, performance-based tasks, portfolio documentation, or curriculum-embedded approaches in familiar settings). The remaining 14 tools served primarily as criterion, comparison, or supplementary measures (e.g., standardized teacher- or parent-report questionnaires, standardized diagnostic instruments) and are noted for context but not cataloged as primary authentic assessment tools.

Nineteen studies (57.6%) employed two or more assessment tools relevant to SED, while 14 studies (42.4%) focused on a single primary tool. Three tools appeared across multiple studies: the Individualized Classroom Assessment Scoring System (inCLASS) (Downer, 2010 [26]; Alamos, 2022 [28]; Sutherland, 2018 [27]); the Minnesota Preschool Affect Checklist—Revised/Shortened (MPAC-R/S) (Sahin Asi, 2019 [45]; Herndon, 2013 [34]); and the Penn Interactive Peer Play Scale (PIPPS/PIPPS-T) (Gagnon, 2004 [23]; Bulotsky Shearer, 2010 [30]). Teaching Strategies GOLD was examined in two studies (Lambert, 2015 [36]; Qiu, 2021 [40]), one addressing the original edition and one the Birth through Third Grade edition.

#### 5.3.2. Classification by Type of Authentic Assessment

Tools were classified according to the assessment type taxonomy defined in the review protocol. Table 6 presents the distribution.

Naturalistic observation was the dominant assessment approach, employed in 23 of the 33 studies (69.7%). Within this category, tools varied considerably: some were direct observational coding systems applied by trained researchers in real-time or from video recordings (e.g., MPAC-R/S, inCLASS, TCIDOS, APIOS, CASPA, video observation coding in Kurki, 2015 [51]; peer engagement coding in Hanish, 2021 [39]), while others were teacher-completed rating scales that drew on teachers’ accumulated naturalistic observations of children’s behavior in classrooms (e.g., ASPI, SCBE, ERC, PSBS-T, Alle Med, LTR, SES). This distinction has important implications for the degree to which tools capture real-time, moment-to-moment behavior versus retrospective informant perceptions, a point that multiple reviewer comments flagged (see Section 6 below).

Play-based assessment tools were used in 10 studies (30.3%), encompassing both semi-structured play protocols administered by researchers or clinicians (e.g., APS-P, TPBA, DJAA, Gift Wrap Task) and observational measures of free play completed by teachers or researchers in classroom contexts (e.g., PIPPS, Revised Knox, Teacher Impressions Scale, ToPS, DPPA).

Performance-based assessment was less common (4 studies, 12.1%), represented by the Preschool Self-Regulation Assessment (PSRA; Rimm-Kaufman, 2009 [29]), the Ounce Scale (Meisels, 2010 [31]), the Dynamic Assessment of Self-regulated Learning in Preschool (Silva Moreira, 2022 [47]), and a peer nomination sociometric procedure (Hernández, 2017 [38]).

Portfolio/curriculum-embedded assessment was represented exclusively by Teaching Strategies GOLD and its expanded edition (Lambert, 2015 [36]; Qiu, 2021 [40]), in which teachers collect documentation (observations, artefacts, video recordings) during naturally occurring daily activities and rate children along developmental progressions.

## 6. Discussion

### 6.1. The Central Tension of the Field Shares

Across the 33 included sources, one overarching dialogue emerges: how to honor the situated, relationship-embedded nature of young children’s social–emotional lives while still producing evidence that is credible, communicable, and actionable for diverse stakeholders. This tension is not new. It echoes longstanding debates in early childhood education between developmental-constructivist and psychometric-accountability traditions [16,58]. What this review reveals, however, is that the conversation appears to be entering a more nuanced phase. Rather than framing authenticity and rigor as a binary, recent scholarship increasingly asks under what conditions, for whom, and through what mechanisms authentic assessment can simultaneously serve formative pedagogical purposes and satisfy systemic demands for accountability. The 0–8 age range sharpens this question because it spans two institutionally distinct worlds: early childhood education and care (ECEC) settings and formal primary schooling, each of which is governed by different policy logic, professional cultures, and expectations regarding what counts as valid evidence of social–emotional competence.

### 6.2. Conceptual Boundaries—What “Authentic” Means and for Whom

One key finding concerns how “authenticity” is defined in the social–emotional domain. Our mapping shows that some sources ground authenticity primarily in ecological context (i.e., assessment occurs during naturally occurring routines and interactions) [8,9], while others foreground participatory epistemology (i.e., children, families, and educators co-construct the meaning of evidence). A smaller but growing cluster highlights cultural authenticity, emphasizing that assessment should not rely only on dominant-culture expectations for emotional expression, self-regulation, or social competence [14,17].

These three framings are related, but they have different implications for tool design, assessor roles, and evidence use. Prior scholarships have done valuable work in articulating each framing independently; what remains less developed is a coherent integrative framework that specifies how ecological, participatory, and cultural dimensions of authenticity interact and where trade-offs arise, when practitioners make moment-to-moment assessment decisions in real classrooms or home-visiting programs. Our review suggests that this integrative theorizing is the logical next step: without it, the field risks producing tools that satisfy one dimension of authenticity while inadvertently compromising another.

These definitional differences also shape assessment practice. For example, a deficit-focused approach may interpret a child’s non-participation in group activities as social withdrawal. A strengths-based approach may view the same behavior as sustained engagement in solitary imaginative play [9]. A relational approach may focus instead on the peer or classroom dynamics surrounding the child. These differences show that definitions of authenticity can influence what assessors notice, how they interpret evidence, and what supports they recommend.

### 6.3. Measurement Architecture: Observation, Portfolio, and Curriculum-Embedded Approaches

A second shared concern is methodological: which formats best capture the dynamic, relational, and context-sensitive nature of social–emotional development in early childhood? Our review identified three dominant formats: structured naturalistic observation systems, portfolio-based documentation, and curriculum-embedded performance tasks. Each has accumulated a distinct evidence base.

Structured observation systems (e.g., those using standardized rubrics applied during free play or routine transitions) have attracted the most psychometric attention [26,27,34]. Existing studies have made important contributions by demonstrating that inter-rater reliability can reach acceptable thresholds when raters receive sustained training. However, this line of work has predominantly focused on the technical properties of the instrument itself, specifically reliability coefficients, factor structures, concurrent validity with norm-referenced scales, while the interactional processes through which observers interpret children’s social–emotional behavior in situ remain comparatively under-examined. In other words, we know a good deal about whether two trained raters agree, but far less about the interpretive reasoning, cultural assumptions, and relational knowledge that shape what an observer notices, selects as salient, and ultimately records. Future research that examines the reasoning processes underlying observer cognition, perhaps drawing on think-aloud protocols or video-stimulated recall, could yield insights that improve not only scoring accuracy but also the pedagogical utility of observation.

This pattern suggests a need to examine not only tools, but also the conditions under which tools are used well. The same observation protocol may function differently depending on the assessor’s training, cultural background, relationship with the child, and familiarity with the classroom context. In this sense, the educator or observer is not simply a neutral user of the instrument. The assessor helps shape what becomes visible as evidence [18,19]. Future research could examine assessor reasoning through methods such as think-aloud protocols, video-stimulated recall, inter-rater discussion analysis, or case-based professional judgment tasks. Such work would extend the psychometric literature by clarifying how observation data are interpreted and translated into pedagogical decisions.

Portfolio and documentation approaches offer a different contribution. They can capture social–emotional development through photographs, learning stories, annotated work samples, teacher notes, and records of children’s participation in everyday routines [31,32]. These approaches are well suited to documenting children’s strengths, relationships, and developmental trajectories over time. They may also create more space for teacher judgment, family input, and children’s voices. However, the reviewed literature provides less evidence on how documentation is selected, interpreted, and evaluated. For example, when a teacher chooses a particular peer interaction as evidence of social competence, the criteria guiding that selection are often implicit. Future studies should examine how educators make these decisions and how documentation practices can remain flexible while still supporting consistency, transparency, and equity.

Curriculum-embedded performance tasks occupy a middle ground, and our review found relatively fewer sources addressing this format in the social–emotional domain specifically. The existing work has usefully shown that embedding assessment within meaningful curricular activities can reduce the artificiality that undermines social–emotional validity [36,40]. Yet most available examples are tightly coupled to a specific curriculum model (e.g., Tools of the Mind, PATHS), raising the question of whether the assessment insights generated are portable across pedagogical approaches. Research that investigates curriculum-embedded social–emotional assessment across diverse program models, including play-based, Reggio-inspired, and culturally specific curricula, would significantly broaden the evidence base.

### 6.4. Multi-Informant Perspectives and the Question of Coherence

A third key finding concerns whose perspectives are included in authentic SED assessment. Our review confirms that most tools and processes center the educator as the primary assessor. A subset of sources involves parent or family input, and an even smaller number attempt to incorporate the child’s own perspective through self-report, drawing, narrative, or play-based elicitation.

Prior research shows that agreement among informants on children’s social–emotional behavior is often low to moderate across both standardized and authentic approaches [45,59]. This body of evidence has rightly been interpreted as supporting multi-informant models. However, the discussion has largely remained at the level of recommending triangulation as a general principle, without providing detailed guidance on the practical mechanisms through which discrepant perspectives are reconciled in authentic assessment contexts. For example, when an educator’s observation suggests a child demonstrates strong emotion regulation during group time, but a parent reports frequent emotional outbursts at home, how should this discrepancy be understood, documented, and used to inform support? Future research should therefore move beyond recommending triangulation as a general principle. More attention is needed to how educators, families, and specialists assemble, compare, and weigh different forms of evidence. Studies should also examine the conditions that support productive dialogue among informants, including time, shared language, cultural responsiveness, and trust. Such work would clarify how multi-informant evidence can be translated into assessment decisions and practical support for children.

### 6.5. Equity, Cultural Responsiveness, and the Politics of “Appropriate” Social–Emotional Behavior

A central and unresolved debate in the field concerns whether authentic assessment, as currently practiced, advances or inadvertently undermines equity for children from historically marginalized communities. Proponents argue that authentic assessment’s attention to context, relationships, and individual trajectories is inherently more equitable than norm-referenced testing. Critics counter that authenticity does not automatically confer cultural responsiveness: observation rubrics may encode dominant-culture expectations for eye contact, emotional expressiveness, or conflict resolution styles; “learning story” templates may privilege linear narrative forms unfamiliar to some cultural communities [15]; and the very constructs targeted such as self-regulation, empathy, social initiative, may carry culturally specific valences.

The reviewed studies acknowledge cultural responsiveness more often as a principle than as an empirical focus. Few studies examined how culture shapes the display, observation, interpretation, or evaluation of children’s social–emotional behavior. This gap is important because culturally responsive assessment requires more than adapting language or applying existing tools to new groups [60]. The field needs detailed empirical work that examines how specific cultural practices, values, and communicative norms mediate the way children’s social–emotional behaviors are displayed, perceived, and evaluated in authentic assessment encounters. Such work would benefit from collaborative research designs that position families and community members as co-designers of assessment tools and co-analysts of assessment evidence.

### 6.6. Developmental Continuity Across the 0–8 Span

Another important gap concerns developmental continuity across the birth-to-eight continuum. Our review reveals a notable institutional and methodological fragmentation: assessment approaches used with infants and toddlers (often relationship-based, dyadic, and conducted in home or small-group settings) differ markedly from those used in pre-kindergarten and early primary classrooms (often group-oriented, teacher-administered, and aligned with learning standards). Few studies explicitly examine transition points, such as home to center-based care, preschool to kindergarten, or kindergarten to early primary school. These transitions are precisely where continuity of social–emotional assessment information may be most important for children.

Previous scholarship has identified the structural barriers to continuity, including different regulatory frameworks, data systems, and professional preparation pathways across the 0–3, 3–5, and 5–8 sectors [2]. What is less well understood is the conceptual challenge of developmental scaling: how to design assessment frameworks that are sensitive to the qualitative transformations in social–emotional functioning that occur across this age range (e.g., from co-regulation to self-regulation, from parallel play to collaborative play, from emotion expression to emotion understanding) while maintaining enough conceptual and procedural coherence to support longitudinal tracking and cross-sector communication. Future work that draws on developmental theory, particularly dynamic systems and relational developmental systems perspectives to inform the design of vertically articulated authentic assessment frameworks would address a significant gap.

### 6.7. Existing Contributions and Future Directions

Taking together, the reviewed literature shows that authentic assessment can support more contextualized understanding of young children’s social–emotional development. The studies included identify a range of feasible approaches, including structured observation systems, play-based assessments, portfolio documentation, learning stories, and curriculum-embedded tasks. Some tools also show evidence of reliability and validity when assessors receive appropriate training [26,36]. At the same time, the review highlights several areas where the evidence base remains limited [61]. These include the integration of ecological, participatory, and cultural dimensions of authenticity; the interpretive processes through which assessors use evidence; mechanisms for reconciling multi-informant perspectives; culturally responsive tool development; continuity across the 0–8 span; and the role of technology in documentation and interpretation (See Table 7).

### 6.8. Synthesis and Implications

The evidence charted in this review suggests, albeit within the descriptive limits of a scoping design, that the first-generation question “Is authentic assessment a viable alternative to standardized testing for young children’s social–emotional development?” has been largely answered in the affirmative. The second-generation questions that now demand attention are more granular and relational: How do the interpretive practices of assessors’ shape what counts as social–emotional competence? Under what institutional and relational conditions do authentic assessment improve the quality of service children receive? How can assessment frameworks honor cultural plurality without retreating into relativism that forecloses shared understanding? And how can developmental continuity be maintained across an age span that is governed by fragmented policy and professional systems?

These questions are complex and cannot be answered by psychometric studies alone, nor by philosophical argument alone. They require methodological diversification, including ethnographic, participatory, design-based, and longitudinal approaches, as well as interdisciplinary dialogue that brings together measurement science, developmental psychology, sociology of childhood, and early childhood pedagogy. The landscape map from this review can guide researchers and practitioners working to advance this next phase of inquiry.

### 6.9. Implications for Practice and Policy

For educators, the landscape map assembled in this review offers a practical starting point for tool selection. The classification rules described in Section 4.4 can help practitioners judge whether a candidate instrument documents naturally occurring behavior in familiar routines, draws on familiar informants, and grounds scoring in documented observation, and Table 4 and Table 6 support matching tools to setting and age band. Equally important, the findings on interpretive practice (Section 6.3 and Section 6.4) suggest that professional preparation should extend beyond administration fidelity to the interpretive dimensions of assessment—what observers are directed to notice, how evidence is weighed, and how strengths-based documentation can counterbalance deficit-oriented readings of children’s behavior.

For clinicians and early interventionists, the subset of tools validated with children with disabilities (Section 5.2.5; Table 5) identifies instruments with evidence of applicability beyond typically developing populations, while the low-to-moderate cross-informant agreement documented in Section 6.4 argues for structured routines—rather than ad hoc judgment—for assembling, comparing, and reconciling discrepant evidence from teachers, families, and specialists.

For policymakers, three investment priorities follow directly from the gaps identified in this review: (a) directing tool-development and validation funding toward the under-served segments of the 0–8 span (infants and toddlers, and the kindergarten–primary transition) and toward world regions absent from the current evidence base; (b) resourcing cultural adaptation as construct-level, co-designed validation with local communities rather than translation of existing instruments; and (c) supporting vertically articulated assessment frameworks that maintain continuity of social–emotional information across the institutional transitions of the birth-to-eight continuum. Together, these priorities indicate how the identified gaps can guide the development of culturally sensitive assessment tools applicable across diverse educational contexts.

### 6.10. Limitations

The present review has several limitations that should be acknowledged. First, inclusion was restricted to English-language publications, which necessarily limits the geographic scope of the evidence base and may have excluded relevant scholarship from non-Anglophone contexts. Second, the search was limited to peer-reviewed journal articles; grey literature sources (technical manuals, government reports, conference proceedings) were included only where the tool described was not captured by any peer-reviewed source, meaning that some practitioner-developed tools may be under-represented. Third, the decision to focus specifically on authentic assessment as opposed to the broader category of formative or classroom-based assessment was theoretically motivated but may have excluded work that shares conceptual commitments with authentic assessment while using different terminology.

Finally, our review is bound by the inclusion criteria we set. By focusing on children aged 0–8, we captured a developmentally and institutionally diverse span. However, we were unable to examine in depth how authentic social–emotional assessment connects to or diverges from approaches used with older children and adolescents. Similarly, by requiring that sources explicitly address “authentic” assessment (or closely related terms), we may have excluded relevant work on formative assessment, classroom-based assessment, or teacher judgement that shares conceptual commitments with authentic assessment but uses different terminology. Future reviews that adopt a broader terminological net, or that focus specifically on one institutional segment of the 0–8 continuum (e.g., infant–toddler care, pre-kindergarten, or early primary), could complement the panoramic view offered here. We may have excluded relevant scholarship from non-Anglophone contexts, including well-established traditions of formative and shared assessment developed in Spanish-language scholarship; the geographic gaps reported here should therefore be read as gaps in the English-language peer-reviewed literature rather than as an absence of relevant practice in unrepresented regions.

## 7. Conclusions

This scoping review has produced the first systematic, theoretically grounded landscape map of authentic social–emotional development (SED) assessment tools for children aged 0–8 years. Conceptually, the review identifies that “authenticity” is operationalized across three partially overlapping dimensions: ecological, participatory, and cultural. Those dimensions are rarely theorized in an integrated manner. The field has convincingly established that authentic approaches are both feasible and preferable to decontextualized testing for the SED domain. The critical next agenda concerns practice-level mechanisms: how assessors interpret evidence, how multi-informant perspectives are reconciled, how cultural plurality is honored, and how developmental continuity can be maintained across the fragmented 0–8 institutional landscape. Methodological diversification through combining ethnographic, participatory, design-based, and longitudinal approaches with sustained interdisciplinary dialogue are required to advance this agenda. The integrated sociocultural and interpretive assessment framework applied here may offer a transferable analytic model for future reviews in adjacent domains of early childhood assessment, subject to the descriptive limits of the scoping methodology.

## Figures and Tables

**Table 1:** PCC Framework.

Element	Description
Population	Children aged 0–8 years (infants, toddlers, preschoolers, kindergarteners, and early primary-grade children)
Concept	Authentic assessment tools (naturalistic observation, play-based assessment, portfolio, performance-based assessment, curriculum-based measurement) targeting social–emotional development (self-awareness, self-regulation, social awareness, relationship skills, responsible decision-making, and related constructs)
Context	Any setting (home, childcare, preschool, primary school, clinic) globally; published literature in English, 2006–2026

**Table 3:** Geographic Distribution of Included Studies.

Country	*n*	%	Studies
United States	19	57.6	Gagnon 2004 [23]; Rimm-Kaufman 2009 [29]; Bulotsky-Shearer 2010 [30]; Downer 2010 [26]; Meisels 2010 [31]; Moreno 2011 [32]; Razza 2012 [33]; Herndon 2013 [34]; Gower 2014 [35]; Lambert 2015 [36]; Alquraini 2015 [37]; Hernández 2017 [38]; Sutherland 2018 [27]; Hanish 2021 [39]; Qiu 2021 [40]; Alamos 2022 [28]; Mathis 2022 [41]; Lasch 2023 [42]; Boise 2024 [43]
Turkey	2	6.1	Uyanık 2018 [44]; Sahin Asi 2019 [45]
Portugal	2	6.1	Santos 2020 [46]; Silva Moreira 2022 [47]
Italy	1	3.0	Delvecchio 2015 [48]
New Zealand	1	3.0	Ahmad 2019 [49]
Jordan	1	3.0	Hamaidi 2021 [50]
Finland	1	3.0	Kurki 2015 [51]
Sweden	1	3.0	Sedem 2022 [52]
Switzerland	1	3.0	Jaggy 2020 [53]
United Kingdom	1	3.0	Wimpory 2007 [54]
Israel	1	3.0	Bauminger-Zviely 2021 [55]
Canada	1	3.0	Hughes 2014 [56]
Norway	1	3.0	Giske 2018 [57]

**Table 4:** Distribution of Studies by Target Age Band.

Age Band	*n*	%	Representative Studies
Infants and toddlers (0–2 years)	6	18.2	Meisels 2010 [31] (0–42 mo); Moreno 2011 [32] (0–38 mo); Razza 2012 [33] (0 cohort); Lasch 2023 [42] (8–15 mo); Giske 2018 [57] (30–33 mo); Ahmad 2019 [49] (from 9 mo)
Preschool (3–5 years)	25	75.8	Uyanık 2018 [44]; Sahin Asi 2019 [45]; Gagnon 2004 [23]; Delvecchio 2015 [48]; Bulotsky-Shearer 2010 [30]; Downer 2010 [26]; Santos 2020 [46]; Alamos 2022 [28]; Hamaidi 2021 [50]; Herndon 2013 [34]; Hanish 2021 [39]; Boise 2024 [43]; Mathis 2022 [41]; Sedem 2022 [52]; Jaggy 2020 [53]; Alquraini 2015 [37]; Sutherland 2018 [27]; Wimpory 2007 [54]; Bauminger-Zviely 2021 [55]; Gower 2014 [35]; Rimm-Kaufman 2009 [29]; Hughes 2014 [56]; etc.
Kindergarten–Primary (5–8 years)	6	18.2	Kurki 2015 [51] (6–9 yr); Silva Moreira 2022 [47] (5 yr 3 mo –7 yr 6 mo); Hernández 2017 [38] (K, 5–6 yr); Rimm-Kaufman 2009 [29] (K); Hughes 2014 [56] (K, ~5 yr); Hamaidi 2021 [50] (K, 5.5 yr)
Broad span (birth–kindergarten)	3	9.1	Lambert 2015 [36] (0–71 mo); Qiu 2021 [40] (0–K); Ahmad 2019 [49] (9 mo to 4.5 yr)

Note: Totals exceed 33 because some studies span multiple age bands. Throughout this article, studies are referred to by the first author’s last name (e.g., Gagnon).

**Table 5:** Population Characteristics of Included Studies.

Population	*n*	%
Typically developing only	20	60.6
Includes children with disabilities/special needs	7	21.2
Includes at-risk/low SES	8	24.2
Both disability and at-risk	3	9.1

Note: Totals exceed 33 because some studies span multiple population.

**Table 6:** Distribution of Primary Authentic Assessment Tools by Type.

Type of Authentic Assessment	N of Distinct Tools	N of Studies Using This Type	Examples
Naturalistic observation	17	23	MPAC-R/S; ASPI; inCLASS; TCIDOS; APIOS; CASPA; Alle Med; LTR; Child Behavior Coding Scale; PARCHISY; SCBE; ERC (teacher); SES; PSBS-T; Play Observation Scale; peer engagement coding (Hanish); video observation coding (Kurki)
Play-based assessment	9	10	Revised Knox Preschool Play Scale; PIPPS/PIPPS-T; APS-P; Teacher Impressions Scale; ToPS; DPPA; TPBA; Gift Wrap Task; DJAA
Performance-based assessment	4	4	PSRA; Ounce Scale; Dynamic Assessment of Self-regulated Learning; Peer Nomination
Portfolio/Curriculum-embedded	2	2	Teaching Strategies GOLD; Teaching Strategies GOLD B-3rd

Note: Some studies employed tools from more than one type; totals therefore exceed 33. Throughout this article, studies are referred to by the first author’s last name (e.g., Gagnon).

**Table 7:** Existing Contributions and Directions for Advancement in Authentic Assessment of Social–Emotional Development.

Existing Contributions	Directions for Advancement
Authentic assessment is theoretically preferable to standardized testing for the SE domain.	Articulating an integrative framework that specifies how ecological, participatory, and cultural dimensions of authenticity interact in practice.
Various tool formats exist and are feasible.	Investigating the interpretive processes and decision architectures through which practitioners actually use these tools—moving from instrument-level to practice-level analysis.
Multi-informant approaches are recommended.	Examining the mechanisms of perspective reconciliation—how discrepant evidence from educators, families, and children is weighed and translated into pedagogical action.
Cultural responsiveness is normatively important.	Conducting empirical studies of cultural mediation in authentic SE assessment, using collaborative designs that position community members as co-analysts.
The 0–8 span is institutionally fragmented.	Developing vertically articulated assessment frameworks informed by developmental theory that support continuity across transition points.
Technology (e.g., digital portfolios, apps) is increasingly used.	Investigating whether and how digital tools alter the relational and interpretive qualities of authentic assessment, rather than evaluating technology adoption in isolation.

## Data Availability

No new data were created or analyzed in this study. Data sharing is not applicable to this article.

## Supplementary Materials

The following supporting information can be downloaded at: https://www.mdpi.com/article/10.3390/brainsci16080806/s1. Supplementary Table S1: PRISMA-ScR checklist; Supplementary Table S2: Data extraction table of included studies (*N* = 33); Supplementary S1: Full electronic search string and database search procedure.
