# Supplementary material for: Authentic Assessment of Social–Emotional Development in Early Childhood: A Scoping Review
Source: Brain Sci. 2026 Jul 30;16(8):806. doi: 10.3390/brainsci16080806 (PMC13510573; doi:10.3390/brainsci16080806)
Supplement: Supplementary file 1 [file brainsci-16-00806-s001.zip › brainsci-4377574-supplementary.pdf]

Supplementary Table S1 PRISMA-ScR Checklist

Manuscript: *Authentic Assessment of Social-Emotional Development in Early Childhood: A Scoping Review*

Authors: Xia, Y.*; Xu, Y.; Zhu, L.; Ai, J.; Chen, C-c.

Based on: Tricco, A.C. et al. PRISMA Extension for Scoping Reviews (PRISMA-ScR): Checklist and Explanation. *Ann. Intern. Med. 2018, 169, 467–473.*

Legend: **Yes** = Fully reported | **Partial** = Partially reported (needs revision) | **No** = Not reported | **N/A** = Not applicable for scoping reviews

SECTION	ITEM #	PRISMA-ScR CHECKLIST ITEM	REPORTED?	LOCATION	NOTES / COMMENTS
TITLE					
Title	1	Identify the report as a scoping review.	Yes	p. 1	Title includes 'A Scoping Review'.
ABSTRACT					
Structured summary	2	Provide a structured summary that includes (as applicable): background, objectives, eligibility criteria, sources of evidence, charting methods, results, and conclusions.	Yes	p. 1	Structured (Background/Objectives, Methods, Results, Conclusions) in 250 words.
INTRODUCTION					
Rationale	3	Describe the rationale for the review in the context of what is already known. Explain why the review questions/objectives lend themselves to a scoping review approach.	Yes	Sec. 1.1–1.3	Three gaps identified; scoping review rationale explained in Sec. 1.3 and 2.3.
Objectives	4	Provide an explicit statement of the questions and objectives being addressed with reference to their key elements (e.g., population or participants, concepts, and context).	Yes	Sec. 1.4	Three RQs stated with PCC framework (Table 1).
METHODS					
Protocol and registration	5	Indicate whether a review protocol exists; state if and where it can be accessed; and if available, provide registration information, including the registration number.	Partial	Sec. 4.1	Protocol developed a priori but NOT prospectively registered. No registration number available.
Eligibility criteria	6	Specify characteristics of the sources of evidence used as eligibility criteria (e.g., years considered, language, and publication status), and provide a rationale.	Yes	Sec. 4.2	Inclusion/exclusion criteria clearly stated: age 0–8, English, 2006–2026, peer-reviewed, authentic assessment tools, SED constructs.
Information sources	7	Describe all information sources in the search (e.g., databases with dates of coverage and contact with authors to identify additional sources), as well as the date the most recent search was executed.	Yes	Sec. 4.3	Six databases listed. Hand-searching and forward citation tracking described. Date of most recent search explicitly stated.
Search	8	Present the full electronic search strategy for at least 1 database, including any limits used, such that it could be repeated.	Yes	Sec. 4.3	Three concept blocks described; full search string referenced in appendix.
Selection of sources of	9	State the process for selecting sources of evidence (i.e., screening and eligibility)	Yes	Sec. 4.4	Two-stage screening (title–abstract, full text),

SECTION	ITEM #	PRISMA-ScR CHECKLIST ITEM	REPORTED?	LOCATION	NOTES / COMMENTS
evidence		included in the scoping review.			two independent reviewers, third reviewer for discrepancies, Cohen's κ reported.
Data charting process	10	Describe the methods of charting data from the included sources of evidence (e.g., calibrated forms or forms that have been tested by the team before their use, and whether data charting was done independently or in duplicate) and any processes for obtaining and confirming data from investigators.	Yes	Sec. 4.4	Data charting form developed a priori, pilot-tested on 10 studies, independent charting by two reviewers for first 20%, random 10% verification.
Data items	11	List and define all variables for which data were sought and any assumptions and simplifications made.	Yes	Sec. 4.4	Variables listed: bibliographic details, study design, participant characteristics, tool characteristics (age range, SED constructs, format, setting, informant, psychometrics), publication period.
Critical appraisal of individual sources of evidence	12	If done, provide a rationale for conducting a critical appraisal of included sources of evidence; describe the methods used and how this information was used in any data synthesis (if appropriate). [OPTIONAL]	Yes	Sec. 4.5	Descriptive appraisal using adapted COSMIN-informed checklist; rationale provided (characterize evidence, not determine inclusion).
Summary measures	13	Not applicable for scoping reviews.	N/A	—	—
Synthesis of results	14	Describe the methods of handling and summarizing the data that were charted.	Yes	Sec. 4.5	Descriptive quantitative summaries (frequencies, percentages) and thematic narrative synthesis; visual evidence map mentioned.
Risk of bias across studies	15	Not applicable for scoping reviews.	N/A	—	—
Additional analyses	16	Not applicable for scoping reviews.	N/A	—	—
RESULTS					
Selection of sources of evidence	17	Give numbers of sources of evidence screened, assessed for eligibility, and included in the review, with reasons for exclusions at each stage, ideally using a flow diagram.	Yes	Sec. 5.1; Fig. 1	417 records, 288 after deduplication, 60 full-text, 33 included. PRISMA-ScR flow diagram (Figure 1) provided.
Characteristics of sources of evidence	18	For each source of evidence, present characteristics for which data were charted and provide the citations.	Yes	Sec. 5.2; Tables 2–5	Detailed by publication year, geography, study design, age groups, population. Citations provided for each study.
Critical appraisal within sources of evidence	19	If done, present data on critical appraisal of included sources of evidence (see item 12). [OPTIONAL]	Yes	Sec. 4.5	COSMIN-informed appraisal mentioned in Methods but detailed results not presented in a dedicated table/figure in Results.

SECTION	ITEM #	PRISMA-ScR CHECKLIST ITEM	REPORTED?	LOCATION	NOTES / COMMENTS
Results of individual sources of evidence	20	For each included source of evidence, present the relevant data that were charted that relate to the review questions and objectives.	Yes	Sec. 5.2–5.3; Table 6 Appendix Table S2	Tool characteristics presented by type (Table 6). Individual study-level charted data presented in a comprehensive table for all 33 studies.
Synthesis of results	21	Summarize and/or present the charting results as they relate to the review questions and objectives.	Yes	Sec. 5.3; Sec. 6	Results synthesized by RQ: tool landscape (RQ1), authenticity conceptualization (RQ2, in Discussion), contextual gaps (RQ3).
Risk of bias across studies	22	Not applicable for scoping reviews.	N/A	—	—
Additional analyses	23	Not applicable for scoping reviews.	N/A	—	—
DISCUSSION					
Summary of evidence	24	Summarize the main results (including an overview of concepts, themes, and types of evidence available), link to the review questions and objectives, and consider the relevance to key groups.	Yes	Sec. 6.1–6.9	Comprehensive discussion linking findings to RQs; relevance to practitioners, researchers, policymakers addressed.
Limitations	25	Discuss the limitations of the scoping review process.	Yes	Sec. 6.10	Language restriction, grey literature limitation, terminological boundaries, age-range scope discussed.
Conclusions	26	Provide a general interpretation of the results with respect to the review questions and objectives, as well as potential implications and/or next steps.	Yes	Sec. 7	Summary of findings, next-step agenda, methodological recommendations provided.
FUNDING					
Funding	27	Describe sources of funding for the included sources of evidence, as well as sources of funding for the scoping review. Describe the role of the funders of the scoping review.	Partial	Funding section	'This research received no external funding.' Funding sources of included studies not described; funder role statement absent.

Supplementary Table S2. Data Extraction Table of Included Studies (N = 33)

Manuscript: *Authentic Assessment of Social-Emotional Development in Early Childhood: A Scoping Review*

Abbreviations: SED = social-emotional development; ASD = autism spectrum disorder; SES = socioeconomic status; ICC = intraclass correlation coefficient; CFA = confirmatory factor analysis; EFA = exploratory factor analysis; IRT = item response theory; DIF = differential item functioning; CASEL = Collaborative for Academic, Social, and Emotional Learning. Full tool names are provided in the main text (Section 5.3).

Study	Year	Country	Design	Age Group	Population	Primary Assessment Tool	Assessment Type	Assessor	Psychometrics Reported	SED Domains	Additional Tools
[23] Gagnon	2004	USA	Quantitative	Preschool (50–66 mo)	At-risk (low SES)	Penn Interactive Peer Play Scale (PIPPS)	Play-based	Teacher; Parent	Construct validity (EFA, 3-factor); concurrent validity with SSRS	Self-regulation; Social interaction; Emotional expression; Broad SED	Vineland SEEC
[54] Wimpory	2007	UK	Quantitative	Preschool (24–49 mo)	Children with ASD	CASPA	Naturalistic observation	Researcher	ICC reported; validity established	Social interaction; Emotional expression; Broad SED	N/A
[29] Rimm-Kaufman	2009	USA	Quantitative	Kindergarten	Typically developing	PSRA	Performance-based	Researcher	α reported; convergent validity	Self-regulation	None
[30] Bulotsky-Shearer	2010	USA	Quantitative	Preschool	At-risk (Head Start)	ASPI	Naturalistic observation	Teacher	α = .74–.91; CFA supported	Social interaction; Emotional expression; Broad SED	PIPPS-T
[26] Downer	2010	USA	Quantitative	Preschool (3–5 yr)	Typically developing	inCLASS	Naturalistic observation	Researcher	ICC = .83–.92; CFA; concurrent validity	Social interaction; Self-regulation; Teacher/Peer interactions	None
[31] Meisels	2010	USA	Quantitative	0–42 months	Typically developing	Ounce Scale	Performance-based	Teacher	α = .80–.95; concurrent validity	Broad SED	None
[32] Moreno	2011	USA	Quantitative	0–38 months	At-risk	Learning Through Relating (LTR)	Naturalistic observation	Teacher	α = .88–.93; test–retest r = .74–.79	Social interaction; Emotional expression	None
[33] Razza	2012	USA	Quantitative	Infants (0 cohort)	Typically developing	Gift Wrap Task	Play-based	Researcher	Validity reported	Self-regulation; Delay of gratification	CBCL
[34] Herndon	2013	USA	Quantitative	Preschool (3–5 yr)	Typically developing	MPAC-R/S	Naturalistic observation	Researcher	ICC = .86 avg; subscale ICCs .75–.93	Self-regulation; Social interaction; Emotional expression; Broad SED	SCBE-30; PLBS; STRS
[35] Gower	2014	USA	Quantitative	Preschool (48–67 mo)	Typically developing	Naturalistic observation coding (aggression)	Naturalistic observation	Researcher	ICC reported	Self-regulation; Emotional expression; Broad SED	PSBS-T
[56] Hughes	2014	Canada	Quantitative	Kindergarten (M = 64 mo)	Typically developing	Play Observation Scale	Play-based	Researcher	ICC reported	Self-regulation; Emotional expression; Broad SED	STRS
[37] Alquraini	2015	USA	Quantitative	Under 5 yr (11–54 mo)	Children with disabilities	TPBA (Linder, 1993)	Play-based	Not reported	Validity/reliability reported	Emotional expression	None
[48] Delvecchio	2015	Italy	Quantitative	Preschool (4–5 yr)	Typically developing	APS-P	Play-based	Researcher	α = .82–.87; convergent validity	Self-regulation; Social interaction; Emotional expression; Broad SED	PSBS-T
[51] Kurki	2015	Finland	Qualitative	6–9 years	Typically developing	Video observation coding (researcher-developed)	Naturalistic observation	Researcher	Inter-rater agreement reported	Self-regulation; Social interaction	MASCS
[36] Lambert	2015	USA	Quantitative	0–71 months	At-risk; Disabilities	Teaching Strategies GOLD	Portfolio/ Curriculum-	Teacher	α = .84–.94; concurrent	Self-regulation; Social interaction; Emotional	None

Study	Year	Country	Design	Age Group	Population	Primary Assessment Tool	Assessment Type	Assessor	Psychometrics Reported	SED Domains	Additional Tools
							embedded		validity	expression	
[38] Hernández	2017	USA	Quantitative	Kindergarten (5–6 yr)	Typically developing	Naturalistic observation (emotion expression)	Naturalistic observation	Researcher	ICC reported	Self-regulation; Emotional expression	Peer Nomination
[57] Giske	2018	Norway	Quantitative	Toddlers (30–33 mo)	Typically developing	Alle Med	Naturalistic observation	Teacher	α reported; validity established	Social interaction; Emotional expression; Broad SED	Early Years Movement Skills Checklist
[27] Sutherland	2018	USA	Quantitative	Preschool (3–5 yr)	At-risk; Disabilities	inCLASS	Naturalistic observation	Researcher	ICC = .80–.90; CFA validated	Social interaction; Self-regulation; Broad SED	TCIDOS
[44]Uyanık	2018	Turkey	Quantitative	Preschool (48–72 mo)	Typically developing	Revised Knox Preschool Play Scale	Play-based	Researcher	α = .80 (Turkish adaptation)	Self-regulation; Emotional expression; Broad SED	ERC; SCBE-30
[35]Ahmad	2019	New Zealand	Quantitative	9 mo – 4.5 yr	Typically developing	Socio-Emotional Competence Index (composite)	Naturalistic observation	Parent; Researcher	Composite reliability reported	Self-regulation; Social interaction; Emotional expression; Decision-making; Broad SED	PSRA Assessor Report
[45] Sahin Asi	2019	Turkey	Mixed-methods	Preschool	Typically developing	MPAC-R/S	Naturalistic observation	Researcher	ICC = .86–.95	Self-regulation; Social interaction; Emotional expression	None
[53] Jaggy	2020	Switzerland	Quantitative	Preschool (3–4 yr)	Typically developing	Tools of the Play Scale (ToPS)	Play-based	Researcher	ICC reported; validity with ToM	Social interaction; Emotional expression; Social-cognitive skills	DPPA
[46] Santos	2020	Portugal	Quantitative	3-year-olds	Typically developing	Social engagement coding	Naturalistic observation	Researcher	ICC reported	Social engagement	Behavioral attributes coding
[55] Bauminger-Zviely	2021	Israel	Qualitative	Preschool (3–6 yr)	Children with ASD	APIOS	Naturalistic observation	Researcher	ICC reported; content validity	Social interaction; Emotional expression; Broad SED	None
[50] Hamaidi	2021	Jordan	Quantitative	Kindergarten (M = 5.5 yr)	Typically developing	SCBE-PE	Naturalistic observation	Teacher	α reported; validity established	Self-regulation; Social interaction; Emotional expression; Broad SED	ERC
[39] Hanish	2021	USA	Quantitative	Preschool (3–5 yr)	Typically developing	Peer engagement coding	Naturalistic observation	Researcher	ICC reported	Social interaction; Emotional expression; Broad SED	Teacher-rated Social Competence
[40] Qiu	2021	USA	Quantitative	0 yr – K	Typically developing	Teaching Strategies GOLD (B-3rd)	Portfolio/ Curriculum-embedded	Teacher	α reported; DIF analysis; IRT	Broad SED	None
[28] Alamos	2022	USA	Quantitative	Preschool (M = 4.39 yr)	At-risk	inCLASS	Naturalistic observation	Researcher	ICC reported; CFA validated	Self-regulation; Social interaction; Emotional expression; Broad SED	ASPI
[42] Lasch	2022	USA	Quantitative	Infants (8–15 mo)	Typically developing	DJAA	Play-based	Researcher	ICC reported; convergent validity	Joint attention	None
[41] Mathis	2022	USA	Quantitative	Preschool (3–4 yr)	Typically developing	SES	Naturalistic observation	Teacher	α reported; concurrent validity	Self-regulation; Broad SED; Attention	PBRS
[52] Sedem	2022	Sweden	Quantitative	Preschool (3–5 yr)	Children with disabilities	Teacher Impressions Scale	Play-based	Teacher	α reported; validity established	Social interaction; Emotional expression	None
[47] Silva Moreira	2022	Portugal	Quantitative	5y3m – 7y6m	Typically developing	Dynamic Assessment of Self-regulated Learning	Performance-based	Researcher	ICC reported; construct validity	Self-regulation	None
[43] Boise	2024	USA	Cross-lagged panel	Preschool (39–54 mo)	At-risk; Disabilities	Child Behavior Coding Scale	Naturalistic observation	Researcher	ICC reported; temporal stability	Self-regulation; Social interaction; Emotional expression; Broad SED	PARCHISY

Supplementary Materials S1

Full Electronic Search String and Database Search Procedure

S.1 Overview

This appendix reports the complete electronic search string used in this scoping review and a worked, reproducible example of how the string was entered and adapted in a single bibliographic database. The search was designed to follow the PRISMA-S recommendations for reporting literature searches and was developed in consultation with a research librarian. It was first built and pilot-tested in PsycINFO (via EBSCOhost) and then translated to each of the remaining databases, with field tags and syntax adjusted to the requirements of each platform.

Databases searched (n = 6): PsycINFO, ERIC, and Education Source (via EBSCOhost); MEDLINE (via PubMed); Scopus; and Web of Science (Core Collection).

Date range: January 2006 to January 2026. Language: English. Publication type: peer-reviewed journal articles.

S.2 Full Search String (Single Line)

The complete string, with the three blocks joined by AND, was entered as follows. This generic form was adapted to each platform’s field tags and syntax (see Section B.6).

(("authentic assessment*" OR "performance based assessment") OR ("assessment*" AND "authent*") OR ("performance assessment" OR "naturalistic observation" OR "play-based assessment" OR "play based assessment" OR "portfolio")) AND (child* OR infant* OR toddler* OR kid* OR preschool* OR kindergarten* OR "early childhood" OR primary) AND (("social*" AND "emotional*" AND "develop*") OR ("social emotional" OR "socialemotional" OR "selfregulation" OR "self-control" OR "responsible decision-making" OR "relationship skills" OR "social competence"))

S.3 Worked Example: Searching One Database (PsycINFO via EBSCOhost)

PsycINFO (via the EBSCOhost interface) was the database in which the strategy was first built and pilot-tested. The basic steps below show exactly how the string was entered and refined. The same logic was then carried over to the other databases.

Step 1.Open the database and select Advanced Search. Log in through your institution, open EBSCOhost, choose PsycINFO, and click “Advanced Search” to access the multi-line search builder and field selectors.

Step 2.Enter each block on its own line and set the search field. Paste Block 1 into the first row, Block 2 into the second, and Block 3 into the third. For each row, set the field selector to TI Title, AB Abstract, and KW/SU Keywords (in EBSCOhost this can be entered directly with the field tag, e.g., TI, AB, or SU).

Row 1 (Block 1): TI (...) OR AB (...) OR SU (...)
Row 2 (Block 2): TI (...) OR AB (...) OR SU (...)
Row 3 (Block 3): TI (...) OR AB (...) OR SU (...)

Step 3.Combine the three rows with AND. Set the operator between each row to AND so that a record must satisfy all three concept blocks. EBSCOhost will display the combined query in the search box.

Step 4.Apply limiters. Restrict the results to: publication date January 2006–January 2026; language = English; and source type = Peer-Reviewed / Academic Journals. (Do not apply an age limiter at this stage — see the note below.)

Step 5.Run the search and record the yield. Execute the search and note the number of records returned. Save the exact query string, the limiters applied, the interface/platform, and the date the search was run, for reporting in the PRISMA-S checklist.

Step 6.Export the results. Export all records (RIS or the platform's direct-export option) and import them into the reference/screening manager (e.g., Covidence) for de-duplication and screening.

Step 7.Translate to the next database. Repeat Steps 1–6 in each remaining database, substituting the platform-appropriate field tags and syntax (Section B.6). Keep the conceptual logic (three blocks joined by AND) identical across databases.
